# [Expression of Concern] Endosialin/CD248 may be a potential therapeutic target to prevent the invasion and metastasis in osteosarcoma

**DOI:** 10.3892/ol.2026.15744

**Published:** 2026-07-03

**Authors:** Yumiko Kondo, Kanya Honoki, Shingo Kishi, Shiori Mori, Rina Fujiwara-Tani, Shinji Tsukamoto, Hiromasa Fujii, Hiroki Kuniyasu, Yasuhito Tanaka

Oncol Lett 23: 42, 2022; DOI: 10.3892/ol.2021.13160

Following the publication of the above paper, it has been drawn to the Editor's attention by a concerned reader that, regarding the photomicrographs shown in Fig. 3F on p. 6, the ‘SaOS2/MORAb-004’ and ‘SaOS2/FN+MORAb-004’ data panels, and the ‘U2OS/MORAb-004’ and the ‘U2OS/FN’ data panels, both exhibit overlapping sections, such that these data panel pairings, which were intended to show the results of differently performed experiments, have apparently been derived from the same original sources.

The authors have been contacted by the Editorial Office to offer an explanation for the apparent anomalies in the presentation of the data in this paper, and we are awaiting their response. Owing to the fact that the Editorial Office has been made aware of this potential issue surrounding the scientific integrity of this paper, we are issuing an Expression of Concern to notify readers of this potential problem while the Editorial Office continues to investigate this matter further.

